# Genetical genomics of quality related traits in potato tubers using proteomics

**DOI:** 10.1186/s12870-018-1229-1

**Published:** 2018-01-23

**Authors:** Animesh Acharjee, Pierre-Yves Chibon, Bjorn Kloosterman, Twan America, Jenny Renaut, Chris Maliepaard, Richard G. F. Visser

**Affiliations:** 1 Graduate School Experimental Plant Sciences, Wageningen, The Netherlands; 2 Plant Breeding, Wageningen University and Research, PO Box 386, 6700 AJ Wageningen, The Netherlands; 3 Institute of Cancer and Genomic Sciences, Centre for Computational Biology, University of Birmingham, Birmingham, B15 2TT UK; 4 Institute of Translational Medicine, University Hospitals Birmingham NHS Foundation Trust, Birmingham, B15 2TT UK; 5 Centre for BioSystems Genomics, P.O. Box 98, 6700 AA Wageningen, The Netherlands; 6 Business unit BiosciencesWageningen University and Research, P.O. Box 16, 6700 AA Wageningen, The Netherlands; 7 Centre de Recherche Public - Gabriel Lippmann Department of Environment and Agrobiotechnologies (EVA) 41, rue du Brill, L-4422 Belvaux, Luxembourg; 8 Present address: Keygene NV, PO Box 216, 6700 AE Wageningen, The Netherlands

**Keywords:** Genetical genomics, Proteomics, Protein QTL, Potato quality traits

## Abstract

**Background:**

Recent advances in ~omics technologies such as transcriptomics, metabolomics and proteomics along with genotypic profiling have permitted the genetic dissection of complex traits such as quality traits in non-model species. To get more insight into the genetic factors underlying variation in quality traits related to carbohydrate and starch metabolism and cold sweetening, we determined the protein content and composition in potato tubers using 2D–gel electrophoresis in a diploid potato mapping population. Upon analyzing we made sure that the proteins from the patatin family were excluded to ensure a better representation of the other proteins.

**Results:**

We subsequently performed pQTL analyses for all other proteins with a sufficient representation in the population and established a relationship between proteins and 26 potato tuber quality traits (e.g. flesh colour, enzymatic discoloration) by co-localization on the genetic map and a direct correlation study of protein abundances and phenotypic traits. Over 1643 unique protein spots were detected in total over the two harvests. We were able to map pQTLs for over 300 different protein spots some of which co-localized with traits such as starch content and cold sweetening. pQTLs were observed on every chromosome although not evenly distributed over the chromosomes. The largest number of pQTLs was found for chromosome 8 and the lowest for chromosome number 10. For some 20 protein spots multiple QTLs were observed.

**Conclusions:**

From this analysis, hotspot areas for protein QTLs were identified on chromosomes three, five, eight and nine. The hotspot on chromosome 3 coincided with a QTL previously identified for total protein content and had more than 23 pQTLs in the region from 70 to 80 cM. Some of the co-localizing protein spots associated with some of the most interesting tuber quality traits were identified, albeit far less than we had anticipated at the onset of the experiments.

**Electronic supplementary material:**

The online version of this article (10.1186/s12870-018-1229-1) contains supplementary material, which is available to authorized users.

## Background

Potato *(Solanum tuberosum* L.*)* is one of the important food crop consumed worldwide. It is vegetatively propagated by means of tubers which develop from underground stems called stolons that under favourable conditions enlarge and increase in size and shape to form tubers. The active growth and development of tubers is accompanied by important changes in the physiology and genetic regulation that lead to large depositions of starch and storage proteins [1, 2]. The nutritional and industrial value of the tubers is mainly from their carbohydrate content which comprises 80% starch along with nutritionally important concentrations of essential amino acids and Vitamin C [3] considering the large amount of storage proteins of the tubers, a proteomics approach was chosen as a suitable way to study potato for specific tuber quality traits.

A number of publications has recently appeared on potato proteomics although research in this area is still limited and fragmented Examples are: tuber mitochondrial proteome [4], abiotic stress response [5], proteomic biomarkers [6], starch potatoes for drought tolerance [7] and sucrose and the raffinose family of oligosaccharides [8].

The proteomics study in the present publication was additional to transcriptomic and metabolomics studies already performed using profiling of the same tubers [9, 10].

Quantitative trait locus analysis has been applied to levels of gene expression enabling the identification of genomic loci controlling the observed variation in gene expression (eQTLs). This approach was called ‘genetical genomics’ [11–13]. Similar approaches can be followed for data derived from other ‘~omics’ technologies such as proteomics (resulting in pQTLs, protein QTLs) and metabolomics (mQTLs, metabolite QTLs) [14, 15].

Although transcriptomic and metabolomics studies are much more applied, proteomic studies were undertaken in a variety of other crops including barley [16], soybean [17, 18], pea [19] and brassica [20, 21].

In this study, we generated proteomics data from a well-studied diploid potato mapping population (here denoted as C x E) using 2D–DIGE (two-dimensional difference gel electrophoresis). We mapped the variation in protein levels by treating these levels as quantitative traits in a QTL analysis. In addition, we performed a QTL analysis for several quality related traits (including starch content and cold sweetening), to study co-location of protein QTLs and phenotypic QTLs. These are traits for which in many cases there was no prior knowledge with respect to which genes might regulate or determine these traits. Identifying metabolites or proteins may then help in getting an idea about the potential genes involved. We identified pQTL and phenotypic QTL (phQTL) hotspot areas [22] across the potato genome and detected pQTLs that co-localized with phenotypic QTLs. Through identification of the proteins and combining the protein QTL (pQTL) results with QTLs from phenotypic traits (phQTL) we hoped to acquire knowledge about the genes and/or proteins which are controlling the variation in quantitative phenotypic traits. In addition, we studied the direct correlation between the phenotypic traits and the protein intensities. This approach offers a tool for plant breeders to get insight into the genetics of complex traits which primarily depend on protein content, constitution, and/or expression. We made a first attempt for the identification of some of these co-localizing protein spots.

## Methods

### Plant materials

A diploid potato (*Solanum tuberosum* L.) mapping population C (USW5337.3) X E (77.2102.37) was used, consisting of 98 progeny individuals plus parents [23]. The genotypes were grown in the field in 2002 and 2003 and the tubers were harvested [24]. All clones were grown in Wageningen, The Netherlands during the normal potato growing season (April–September). For each genotype, all tubers were collected from three plants and representative samples were either used for phenotypic analyses or mechanically peeled and immediately frozen in liquid nitrogen before being ground into a fine powder and stored at − 80 °C for subsequent proteomic analysis.

### Phenotypic analyses

Different quality traits were considered in the phQTL study. A detailed list of phenotypic traits that were assessed can be found in the Additional file 1: Table S1. In this study, we focused on 26 quality traits related to starch characteristics (11 traits) and colour and cold sweetening (15 traits). A detailed description of how the different traits were assessed and analyzed in this CxE mapping population can be found in [24].

### Proteomics data generation and processing

#### Protein extraction

Total protein was extracted of each of the parental and progeny clones from approximately 0.5 g of ground tuber material that had been stored at − 80 °C, to which 1 ml of pre-heated (95 °C) lysis buffer (50 mM sodium phosphate buffer pH 7, sucrose (5% *w*/*v*), SDS (4% w/v), DTT (0.3% w/v), PVP-P (10% w/v)) was added. Samples were homogenized for 45 s, placed at 95 °C in a water bath for 1 min and homogenized again (45 s, speed 6.5 m/s). After 3 min at 95 °C in water bath the samples were cooled on ice and centrifuged for 15 min. 4 ml cold acetone (− 20 °C) containing 10 mM DTT was added to the supernatant. This was vortexed vigorously and put at − 20 °C for 1 h. The protein extract was centrifuged for 20 min in a Centricon T42-k (25,000×*g*, 4 °C). The pellet was washed with 4 ml cold acetone (− 20 °C) containing 10 mM DTT twice. After air drying the pellet, the pellet was dissolved in 300 μl TUCCDT buffer (urea 5 M, thio-urea 2 M, C7BzO (2% *w*/*v*), CHAPS (2% w/v), DTT (0.3% w/v), TCEP 2 mM). Protein amount was measured using the RC/DC assay (Biorad, Veenendaal, the Netherlands) using Bovine Serum Albumine (BSA) as standard for the calibration curve.

#### Protein labelling

The proteins were stained using a co-valent attached fluorescent probe using the Difference Gel Electrophoresis (DIGE) technology (GE Healthcare) according to the manufacturer’s protocol. The dye to protein ratios were chosen such that on average a single lysine per protein molecule was labelled using the fluorescent Cy dyes, either Cy2, Cy3 or Cy5. The internal standard was labelled with Cy2 and consists of an equal mixture of protein extracts of 20 randomly chosen samples of the experiment (9 random samples from 2002 and 2003 each and both parents C and E from 2003).

Every 2D–gel contained one sample labelled with Cy3, one labelled with Cy5 and the internal standard labelled with Cy2. The use of internal standard sample labelled with Cy2 on each gel enabled better alignment of gel images and was also used for quantitative normalisation between multiple gels.

#### 2D–electrophoresis

The first dimension electrophoresis was performed using 24 cm immobilized pH gradient strips (GE Healthcare) with a linear pH range from 4 to 7 on an Ettan IPGPhor isoelectric focusing (IEF) system. Cydye labelled samples (total of 150 μg protein) were loaded to the strips diluted in 0.5% IPG buffer (pH 4–7 and pH 3–10, 1:1) and TUCCDT buffer to a volume of 450 μl. The focusing was run for 18 h at 20 °C with the following settings: 3 h 150 V, 3 h 300 V, from 300 V to 1000 V in 6 h, from 1000 V to 10,000 V in 1 h and finally 5 h at 10000 V. After IEF the strips were equilibrated in the dark at room temperature in equilibration buffer (urea 6 M, 50 mM Tris-HCl pH 8.8, glycerol 30% (*v*/v), SDS 2% (*w*/*v*)) containing DTT 1% (w/v) for 15 min and after that in the same buffer (without DTT) with iodoacetamide 2.5% (w/v) for 15 min. The second dimension electrophoresis was run on the Ettan Dalt twelve system on precast 12.5% SDS polyacrylamide slab gel (size: 255x196x1 mm) and buffers from GE Healthcare. Electrophoresis was performed at 1 W/gel for 1 h followed by 1.5 W/gel until bromophenol blue had reached the end of the gel (approximately 17 h) at 15 °C. The separated CyDye-labelled proteins were visualized by scanning with an Ettan Dige Imager (GE Healthcare), using for Cy2 a 480 nm laser and an emission filter of 530 nm, for Cy3 an 540 nm laser and an emission filter of 595 nm and for Cy5 an 635 nm laser and an emission filter of 680 nm.

### Image analysis and data pre-processing

Gel images were analysed with the Decyder software version 7 according to Decyder 2Dv.7 manual (GE Healthcare). The detected spots were then filtered based on spot volume larger than relative value 30,000 to exclude spots that could be just background noise or dust particles. The internal standard in each gel was used to automatically match all images to the reference (the gel with the largest number of detected spots). After that a gel area with saturated spots coming mostly from patatin was excluded because these proteins were at the ceiling level of detection for all samples as these are rather abundant (storage) proteins. To make 2D–spot alignment across the samples a clear image gel was chosen as the master and added to all the gel batches (1 batch is one run of 12 gels). Then these batches were linked to each other by automatic matching in the software program and corrected afterwards manually with the help of setting landmarks (i.e. spots visible in all images). The spot volume ratio to the internal standard of each protein and the individual volume of the spots were calculated and log_10_ transformed. In the QTL analysis the spot volume (intensity) value was used. Each of the proteins are presented by Pro_X where “X” represents consecutive protein numbers, numbered from top to bottom and from left to right starting with number 1 in the top left and ending with number 1643 in the right bottom of the gel.

#### Protein identification

Spots of interest (ie confirming to the absence/presence or varying amounts between different gels and over the two harvests) were excised from gel using the Ettan Spot Picker. We focused on the ones which were leading to a pQTL in both years and to some of the unique ones in a given year. In total attempts were made to isolate protein from around 120 spots. After washing and desalting in 50 mM ammonium bicarbonate/50% *v*/v methanol, followed by 75% v/v ACN, spots were digested with Trypsin Gold (MS grade, Promega, Madison, WI, USA, 8 mg.mL-1 in 20 mM ammonium bicarbonate) using the Ettan Digester robot. Automated MALDI spotting of the samples was carried out with the spotter of the Ettan Spot Handling Workstation. Peptides dissolved in a 50% ACN (v/v) solution containing 0.5% TFA (v/v) (0.7 mL) were spotted on MALDI-TOF disposable target plates (4800, ABSciex, Foster City, CA, USA) prior to the deposit of 0.7 mL of CHCA (7 mg/mL, 50% v/v ACN, 0.1% v/v TFA, Sigma Aldrich, St. Louis, MO, USA). Peptide mass determinations were carried out using the Applied Biosystems 4800 Proteomics Analyzer. Both PMF and MS/MS in reflectron mode analyses were carried out with the samples. Calibration was carried out with a peptide mass calibration kit. Proteins were identified by searching against the NCBI ‘viridiplantae’ database (September 2011) and an EST ‘viridiplantae-eudicots’ database (October 2010) using MASCOT. All searches were carried out using a mass window of 50 ppm for MS and 0.75 Da for MS/MS. The search parameters allowed for carboxyamidomethylation of cysteine as fixed modification, and oxidation of methionine as variable modification. Homology identification was retained with a probability threshold of 95%, all identifications were manually checked.

#### QTL mapping

QTL mapping of protein abundances of the clones in the mapping population was done based on the spot volume ratio to the internal standard (intensity) of the proteins (after transforming the different spots into a quantitative value). QTL analysis of the protein abundances as quantitative traits, was done using the R/qtl library [25]. A genome-wide LOD significance threshold (4.28) was computed using the [26] and was used for all QTL analyses. The data was loaded in R and run through the jittermap function from R/qtl and probabilities of the underlying genotypes were computed using a hidden Markov model, as available in the calc.genoprob function of R/qtl with a step size of 2.5 cM. We performed the “4way” (terminology used in R/qtl for a cross between two heterozygous diploid parents) procedure for simple interval mapping using the Haley-Knott regression method [27]. Significant QTLs (LOD > 4.28) were extracted and the explained variances of these QTLs were computed. For each QTL the following information was reported: start position (cM position where the significance threshold was passed), peak cM position, and stop position (cM position where the LOD score drops under the significance threshold again), start, peak and stop marker, LOD value for the peak marker and the explained variance (R^2^) at the peak position. More detailed information is provided by [28].

The genetic map used in this QTL analyses consisted of 343 markers. This is a modified version of an earlier C x E genetic map [29], with all sequence based SNP markers and extended with additional markers from allele specific hybridization signals using a potato. In order to describe the density of pQTLs and phQTLs over the genome, we calculated numbers of pQTLs or phQTLs using a 10 cM sliding window according to [30].We considered pQTLs to be co-localized with phQTLs if they fell within a 10 cM interval (5 cM to the left and 5 cM to the right) around the peak marker of the phQTL.

#### Correlation analysis of protein abundance and quality traits

Pearson correlations were calculated between the protein abundance values and between protein abundance and quality traits over the clones in the mapping population, and then tested using a *t*-test of each of the correlation coefficients, followed by an FDR (False discovery rate) correction of the *p*-values from these t-tests, using the FDR correction procedure of [31].

### Data availability

In Additional file 1 a list and scoring table is given for the different quality traits measured in the CxE mapping population. In Additional file 2: Table S2 the number of pQTL and their chromosomal locations are depicted. In Additional file 3: Table S3 a summary of the different phenotypic QTL of the various quality traits and their peak positions as well as explained variance is given. Finally, in Additional file 4: Tables S4 and and Additional file 5: Table S5) the colocalization of the phenotypic and protein QTL on the different potato chromsomes in the subsequent growth years 2002 and 2003 is given. All the phenotypic and proteomic data can be found in the Additional file 6.

## Results

**Fig. 1:**
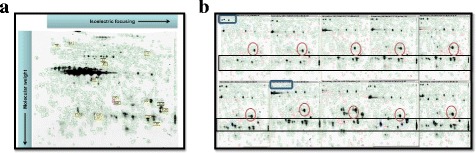
An example of a 2D DIGE gel image (**a**) Different protein spots which are co-localizing with a flesh colour QTL are shown in yellow boxes. The dark protein spots in the middle and left of the gel are the over-abundant patatin proteins. **b** A panel of 10 different gels (in all cases the right bottom quarter is depicted in this figure) showing the patterns and the absence/presence (blue rectangular area), always present in almost similar amounts (round area), as well as several examples of spots with varying quantities over different samples (black rectangular area)

In this study, we generated proteomics data from 2D–DIGE (Difference gel electrophoresis). The patatin protein family (storage proteins in the potato tuber [32] was left out for further analysis because of the overabundance of these proteins, clearly visible as a large block of multiple protein spots in the middle of the gel (Fig. 1a). Initially 1643 unique spots were detected in total over the two harvests of 2002 and 2003. We considered the 2 year harvests to see the consistency and/or difference in the pQTLs. Based on the ANOVA value of the spot volume (below 1%) we did pQTL analysis with 380 protein spots for the 2002 harvest and 320 spots for the 2003 harvest that were measured in all samples. The number of overlapping spots for both years was 255 with an extra 125 observed only for 2002 and an extra 65 unique to 2003. The highest absolute Pearson correlation coefficient among these 255 proteins was 0.98 for both 2002 (between protein nr. 39 and 40) and 2003 (between protein nr. 295 and 297). The analysis was done as described in the materials and methods section by taking a quantitative measure of the different spots (log10 of spot volume) and by analyzing them for the individual 90 genotypes as can be seen from the example in Fig. 1b.

For 2002, 190 significant pQTLs were found for 170 protein spots (113 spots from the 255 common ones, 57 from the protein spots unique for 2002). For the 2003 harvest, we found 173 pQTLs for 154 protein spots (130 from the 255 common ones, 24 spots that were unique for 2003). We found 82 pQTLs that mapped in the same chromosome in both years and out of these 82, 56 pQTLs mapped in the exact same position (identical peak position) in the chromosome across 2 years (Additional file 3: Table S3).

We found 20 proteins for the 2002 harvest with QTLs on two different chromosomes. For 2003, 17 proteins had QTLs in either two or three different chromosomes and those proteins gave 36 pQTLs in total. Out of these 17 proteins, 2 had 3 different pQTLs and 15 proteins had 2 pQTLs each.

Comparing pQTLs from the 2002 and 2003 harvests separately, for 2002 the largest percentage variation explained (R^2^) for a pQTL was 94%, for protein number 429, and this pQTL is mapped to chromosome 7 at 7.4 cM. For 2003, this pQTL maps to the same position with 70% explained variance. The Pearson correlation coefficient in the abundance of this protein between the 2 years, across the potato clones, was 0.81. The QTL with the largest amount of variance explained for the 2003 harvest (74%) was for protein number 1007 and this QTL mapped to chromosome 3. For 2002, this pQTL was found in the same chromosome and the same position and the QTL explained 88% of the variance in protein abundance. The Pearson correlation in the abundance of this protein between the 2 years was 0.75.

In both years the largest number of pQTLs was found for chromosome 8 (41 and 31 pQTLs, for 2002 and 2003, respectively) and the lowest for chromosome number 10 (2 and 3 pQTLs, for 2002 and 2003 respectively).

**Fig. 2:**
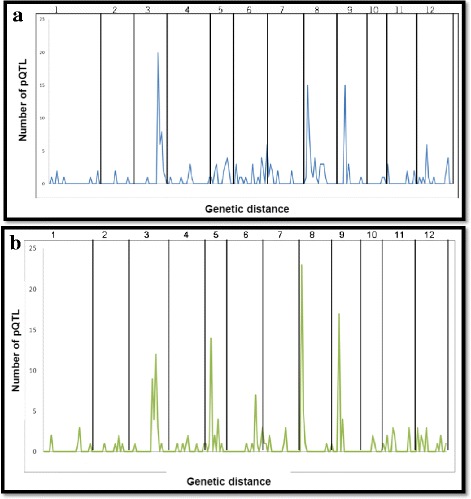
Protein QTL (pQTL) density for proteomics data from 2 years. X-axis represent pQTL genomic location on chromosomes. Y-axis represents pQTL density calculated on a 10 cM sliding window. Chromosomal regions corresponding to the largest number of significant pQTLs are considered pQTL hotspots, on chromosomes 3, 5, 8, 9 for 2 years. **a** Protein QTL (pQTL) density from 2002 data across the genome. **b** Protein QTL (pQTL) density from 2003 data across the genome

To investigate if the pQTLs were evenly distributed across the genome, or clustered in particular regions, we calculated the density of pQTL per cM across the genome using a 10 cM sliding window analysis (Fig. 2a). For the 2002 harvest, four regions had a high pQTL density centering around markers PotSNP749 (position: bch (Chr. 3, 80 cM), PotSNP125 (Chr. 5, 23 cM), PotSNP749 (Chr. 8, 6 cM) and STM3012 (Chr. 9, 16 cM), each having more than 8 pQTL per cM which is much higher than the expected 0.17 pQTLs per cM if the 190 pQTLs were evenly distributed along the 1135 cM genetic linkage map.

For the 2003 harvest a total of 152 pQTLs (88% of the 173 significant pQTLs) are mapped on chromosomes 3, 5, 6, 7, 8, 9, 11 and 12 and the number of pQTLs were 30, 24, 11, 11, 31, 21, 11 and 13 respectively (Fig. 2b). Similar to the 2002 harvest, we observed that hotspot regions of pQTLs are found for chromosome numbers 3, 5, 8 and 9.

### Correlations of protein spots with quality traits

From the Pearson correlation study among protein spots and quality traits 22 protein spots were significantly correlated to quality traits with FDR corrected [31] *p*-value (*p* < 0.05 for the FDR corrected t-test on Pearson correlation). In total 10 protein spots were found significantly correlated with flesh colour. Among these, the highest correlation coefficient was 0.67 for protein number 1129 and the lowest but still significant correlation coefficient 0.34 for protein number 686. The highest correlation coefficient with enzymatic discoloration after 30 min and 3 h were both equal to 0.44 for protein spot number 1129. Four protein spots showed significant correlations to enzymatic discoloration after 30 min and 3 h. Four other protein spots were significantly correlated to starch phosphorylation. The highest correlation coefficient of a protein spot to starch phosphorylation was for protein number 129 (*r* = 0.44).

### Phenotypic QTL (phQTL) analysis

QTLs for the majority of the starch related quality traits such as percentage of amylose and starch gelatinization related traits are mapped to chromosome 2, specifically in the region between 73.7 cM and 80.2 cM (start and end position). A single QTL for flesh colour and enzymatic discoloration is mapped to chromosome 3, in the region between 78.5 cM and 81.4 cM [33]. We did not find any significant phQTLs for the quality traits studied here on chromosomes 4, 7, 9, 11 and 12. Detailed results of the QTL analyses for starch and cold sweetening related traits are presented in Additional file 4: Table S4 & Additional file 5: Table S5).

We focused on co-localizations of phQTLs related to starch traits, (enzymatic) discoloration and cold sweetening and pQTLs for the analyses of 2 years (2002 and 2003). Such co-localizations can be useful to identify proteins involved in the regulation of these phenotypic traits. One other striking observation was that a phenotypic QTL for total protein content [34] on chromosome 3 in the region of 70–80 cM corresponded approximately with 23 different pQTLs (one of the four hot-spot regions of pQTLs).

**Fig. 3:**
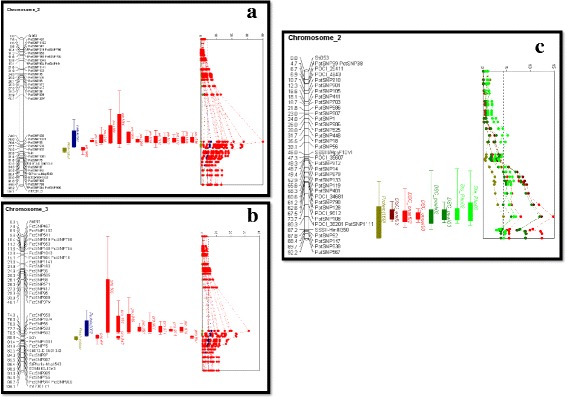
Visualization of protein QTL and phenotypic QTL. **a** Example of the abundance of pQTLs (QTLs are indicated with 2 LOD support intervals) on chromosome 3. pQTLs are shown in red, the QTL for total protein content in blue, for flesh colour in light green. **b** Continuation of example of the abundance of pQTLs on chromosome 3. **c** Another example of the abundance of pQTL on chromosome 2 is shown. QTLs for different quality traits such as differential calorimetry (DSC onset in red, DSC peak in dark green) and starch phosphorylation (in light green) are co-localized with protein number 169

For the 2002 harvest: in chromosome 1, a QTL for starch gravity is co-localized with two proteins (pro_375 and pro_102) between 126.6 cM and 135.0 cM. QTLs for percentage of amylose and starch gelatinization related traits co-localize with a pQTL on chromosome 2 in the region between 73.7 and 80.2 cM. QTLs for flesh colour and enzymatic discoloration (after 5 and 30 min) are co-localized with 14 pQTLs on chromosome 3 between 78.5 and 88.5 cM (Fig. 3a and b). On chromosome 5, phenotypic QTLs for differential scanning calorimetry and chip colour after harvest are co-localized with two pQTLs at 23.6 cM. A QTL for starch-phosphorylation is also co-localized on chromosome 5 with 9 other pQTLs between 40.3 cM to 54.8 cM. A QTL for particle size distribution of the starch is co-localized on chromosome 6, between 56.4 cM to 59.9 cM with 3 pQTLs. A QTL for specific gravity of starch is co-localized with a pQTL on chromosome 8, in the region of 59.2 cM to 67.8 cM.

For the 2003 harvest: in chromosome 1, a QTL for starch gravity is co-localized with a QTL for Protein 1240 between map positions 126.6 and 135.0 cM. QTLs for the percentage of amylose and starch gelatinization related traits are co-localized with QTLs for three protein spots on chromosome 2 between 73.7 and 80.2 cM. QTLs for flesh colour and enzymatic discoloration (after 5 and 30 min) co-localize with QTLs for 31 protein spots on chromosome 3 between 74.0 to 88.5 cM. On chromosome 5, QTLs for differential scanning calorimetry and chip colour after harvest co-localize with QTLs for 15 protein spots in the region between 40.3 and 54.8 cM. A QTL for starch-phosphorylation is also co-localized in chromosome 5 with QTLs of five other protein spots in the region between 40.3 and 51.5 cM. A QTL for particle size distribution of starch is co-localized in chromosome 6, at exactly the same position (56.4 cM) with a QTL for Protein 251 for both the years. We did not find co-localization of any protein QTLs with the QTL for specific gravity of starch for the 2003 harvest. Detailed results are shown in Additional file 4 for the 2002 harvest and in Additional file 5 for the 2003 harvest.

### Protein identification

In a first attempt to try and identify the proteins which were co-localizing with certain phenotypic traits we focused on enzymatic discoloration, flesh colour and some of the starch traits. From the 80 spots of which we were able to isolate protein in a sufficient quantity, we obtained an amino acid sequence for 28 protein spots only. For 17 of these 28, a putative identity could be given based on the NCBI ‘viridiplantae’ database. Based on the protein identification there is sometimes a hit for a specific protein (derived from various plant sources and sometimes it is linked to an identity because the protein fragments do not only resemble but are very homologous to the particular protein). The putative protein identity was converted into a genome sequence based on expressed sequence tag (EST) data (Table 1).

**Table 1:** List of proteins isolated from gel and putatively identified. Full names of the traits are listed in the Additional file 1: Table S1

Protein Nr.	Est	Protein name	Traits associated with the proteins or pQTLs from the proteins in the first column
39	NA	5-lipoxygenase [*Solanum tuberosum*]	Multiple pQTL 2003
62	NA	methionine synthase [Solanum tuberosum]	Enz. Discol5min
64	NA	NA	Flesh Colour
128	gi:58,217,733	gi|108,709,562|gb|ABF97357.1| Lysyl-tRNA synthetase, putative, expressed [*Oryza sativa* Japonica]	Multiple pQTL 2002
129	NA	RecName: Full = Transketolase, chloroplastic; Short = TK; Flags: Precursor	Starch_Phos03
175	gi|10,808,429	gi|8,250,622|emb|CAB93680.1| plastidic phosphoglucomutase [Solanum tuberosum]	Enz. Discol5min
186	NA	NA	Multiple pQTL 2002
180	NA	NA	DSCdH03
200	NA	aminoaldehyde dehydrogenase 2 [*Solanum lycopersicum*]	Enz. Discol5min
196	NA	NA	Enz. Discol5min
193	NA	importin alpha, putative [*Ricinus communis*]	Multiple pQTL 2002
237	NA	chaperonin-60 beta subunit [Solanum tuberosum]	Enz. Discol5min
218	NA	NA	Flesh Colour
280	gi|14,644,452	gi|4,704,766|gb|AAD28260.1|AF131223_1 protein disulfide isomerase homolog [*Datisca glomerata*]	Enz. Discol5min
296	NA	vacuolar H + -ATPase B subunit [*Nicotiana tabacum*]	Top pQTL 2003
339	NA	ATP synthase F1 subunit 1 [Nicotiana tabacum]	Enz. Discol5min
372	NA	beta tubulin [*Capsicum annuum*]	Multiple pQTL 2002
379	NA	ATP synthase beta chain [*Zea mays*]	Starch.grT.2002
411	NA	NA	Enz. Discol5min
964	NA	catalase isozyme 1-like protein [Solanum tuberosum]	Multiple pQTL 2002
1021	gi|53,697,586	gi|161,702,915|gb|ABX76298.1| sexual organ expressed protein [*Nicotiana alata*]	Flesh Colour

We tried to identify more proteins, especially those associated with enzymatic discoloration and flesh colour. These attempts were not very successful although we were able to get an amino acid sequence for some of the proteins. In most cases the putative identities of these proteins did not make immediate sense but in the case of enzymatic discoloration enzyme functions like chaperonin (protein nr 239), protein disulfide isomerase (nr 280), aminoaldehyde dehydrogenase (nr 200), plastidic phosphoglucomutase (nrs 171 & 175) and methionine synthase (nr 62) were retrieved which are among the types of functions which one could imagine that might be involved in this specific pathway. However more research into this area is required.

## Discussion

We did pQTL analysis with 380 proteins for 2002 and 320 proteins for the 2003 harvest separately and phQTL analysis for starch and cold sweetening related traits as well as flesh colour using an integrated linkage map of C x E. The pQTL analysis of the proteomics data resulted in a large number of genetic regions involved in protein abundance. The pQTLs are spread out over all chromosomes but four regions show a larger number of QTLs, so-called “hotspots” [22]. These hotspots contain most probably one causal factor for protein synthesis or regulation which maps to that locus [35]. In other plant species, for example in *Arabidopsis*, similar hotspots were detected after mapping transcripts, protein expression, metabolites, and phenotypic traits [36] These authors reported that the phenotypic variation was mainly due to six hotspots.

In our study, four hotspot regions consistent across the years 2002 and 2003 are found on Chr. 3 near 70–80 cM, on Chr. 5, near 20–30 cM, on Chr. 8, position 6 cM and on Chr. 9, near 10–20 cM. This shows stability of pQTL hotspots across the 2 years. The fact that we find a hotspot for protein content as determined by Werij et al. [34] with over 20 pQTLs may suggest that this concerns an overall regulator of protein synthesis in potato tubers. More research is needed to elucidate this.

In a previous study of expression QTLs (eQTLs) and metabolite QTLs (mQTLs) [9, 10], it was noted that the hotspot areas for expression and metabolites were mainly on chromosome 5 and 11. In the case of pQTL analysis we mainly find pQTL hotspots on chromosomes 3, 5, 8 and 9. This indicates that the genetic regulation of the protein expression and/or content is more likely controlled by specific locations on those chromosomes. Chromosome 5 is in common as a hotspot, for protein QTLs, metabolic and expression QTLs. Also for phenotypic QTLs including some of the agronomical traits, chromosome 5 is a hotspot (data not shown for agronomical traits but see [24] due to pleiotropic effects of maturity or earliness on chromosome 5 (for pleiotropic QTLs for developmental traits see e.g. [37].

The phQTL on chromosome 3 for tuber flesh colour is consistent with earlier finding [33]. Moreover, other reports link the gene beta-carotene hydroxylase with the QTL at this map position [38, 39]. One more gene involved in yellow tuber flesh colour: zeaxanthin epoxidase (Zep) on chromosome 2 [39]. They established this relationship in an association analysis between single nucleotide polymorphism (SNP) haplotypes and flesh colour phenotypes in a large range of diploid and tetraploid potato genotypes. In our analysis only half of the number of genotypes had tubers with yellow flesh colour and the statistical power may not have been enough for detecting this second QTL [38].

In this study we used multi-year (2002 and 2003) protein and phenotypic datasets. For the 2002 harvest, 43% of the pQTLs are mapped to the same chromosome as the harvest of 2003, and, *vice versa*, 47% of the pQTLs from the 2003 harvest are mapped to the same chromosome as those for 2002. For 10 protein spots of the 2002 harvest we found 20 QTLs on two different chromosomes. Out of these 20 QTLs, ten have only a pQTL for the 2003 harvest; the other ten are mapped to the exact same position as for the 2002 harvest. For 2003, QTLs for each of 17 proteins mapped to either two or three different chromosomes or seven out of these proteins have a pQTL only for the 2002 harvest; the ten remaining ones were mapped to the exact same position as for 2003. The proteins which are mapped to the same chromosome across the years show consistency between the years. This is expected as the effects are genetic and the protein abundances are well-correlated across years. This indicates that genotype-by-environment interaction is not very large and that the measurement/technical variation is small in comparison with the genetic variation for these proteins.

### Co-localization of pQTLs and phQTLs

In this study the same mapping population was used to detect phQTLs for carbohydrate related traits and protein traits. We investigated QTL co-localization between phenotypic and protein traits. As an example we have shown co-localization of a flesh colour QTL with QTLs of different protein spots (Fig. 3a and b). A detected QTL indicates a statistical association between a marker locus in that region and the quantitative variation for a given trait segregating in that same population [40]. When QTLs for two different traits co-localize, we could hypothesize the existence of a common locus that contributes to the variation of both traits, or we could consider the association to be due to linkage of different loci. Such hypotheses are useful in the search for candidate genes for phenotypic traits of interest for which most of the genetic basis is unknown.

In the correlation study, we found a protein, protein number 1129 to be positively correlated with flesh colour, and enzymatic discoloration after 30 min and 3 h, with correlation coefficients 0.67, 0.44 and 0.44, respectively. A QTL for this protein was mapped to chromosome 3 at 80.8 cM for both years and co-localizes with the flesh colour QTL as well. From a previous study by [38], it was reported that carotenoids are involved in flesh colour and the beta-carotene hydroxylase (bch) gene plays a major role in flesh colour variation in potato. This gene is indeed located on chromosome 3 and thus it is tempting to speculate that this protein would indeed be BCH but so far we were not able to identify this protein.

## Conclusions

In this paper we demonstrated the use of genetic information from phQTL and pQTL analyses on the one hand and Pearson correlations of phenotypic traits with proteomics data on the other hand. From the QTL analyses, we can identify the map position of the QTLs but associations need not to be from a functional relationship but can also be due to linkage. In correlating phenotypic traits to proteomic data, we find proteins that might be related to the phenotype, but in the absence of genetic information, this correlation could be due to environmental conditions influencing both the phenotypic trait and the protein abundance(s). In some cases however the genetic position and protein position of a particular trait hint to the same chromosomal location and these genes may thus be first candidates to work on in order to prove a connection between trait and pQTL. Combining QTL analysis of protein abundance and of quality traits and correlation analysis among all traits gives us a better understanding about candidate proteins which are linked to the phenotype but also shows which correlations could be due to a genetic association. A similar type of approach was described in the studies of [9, 10, 23] where the authors combined QTL analysis with a prediction of the phenotypes from metabolomics and transcriptomics data using random forest regression.

## Additional files

Additional file 1: Table S1. List of carbohydrate and starch metabolism and cold sweetening related quality traits measured in the CxE population. (DOCX 14 kb)

Additional file 2: Table S2. pQTL analysis summary for 2002 and 2003 harvest. Number of significant protein QTLs is depicted as well as the chromosomes containing the highest and lowest number of pQTLs. (DOCX 12 kb)

Additional file 3: Table S3. Summary of Phenotypic QTL results of the various quality traits, their peak position and explained variance at peak position. (DOCX 14 kb)

Additional file 4: Table S4. Co-localization of phQTLs (starch, colour and cold sweetening related traits) and pQTLs for proteomics data in 2002. (DOCX 13 kb)

Additional file 5: Table S5. Co-localization of phQTLs (starch, colour and cold sweetening related traits) and pQTLs for proteomics data in 2003. (DOCX 25 kb)

Additional file 6: Table S6. Proteomics data from 2002 and 2003 and phenotypic data sets. (XLSX 409 kb)

## Abbreviations

2D–DIGE: Two-dimensional difference gel electrophoresis
ANOVA: Analysis of variance
BSA: Bovine serum albumin
cM: Centimorgan
Cy2: Cyanine 2
Cy3: Cyanin 3
Cy5: Cyanin 5
eQTL: Expression quantitative trait loci
FDR: False discovery rate
LOD: Logarithm of odds
mQTL: Metabolic quantitative trait loci
phQTL: Phenotypic QTL
pQTL: Protein quantitative trait loci
Pro_X: Protein X
QTL: Quantitative trait loci
SNP: Single nucleotide polymorphism
