# Supplementary material for: Genetical genomics of quality related traits in potato tubers using proteomics
Source: BMC Plant Biol. 2018 Jan 23;18:20. doi: 10.1186/s12870-018-1229-1 (PMC5781343; doi:10.1186/s12870-018-1229-1)
Supplement: Supplementary file 1 — List of carbohydrate and starch metabolism and cold sweetening related quality traits measured in the CxE population. (DOCX 14 kb) [file 12870_2018_1229_MOESM1_ESM.docx]

**Additional file 1(Table 1)**: List of carbohydrate and starch metabolism and cold sweetening related quality traits measured in the CxE population.

| Cold sweetening related quality traits | Description | | Scoring | |  |
| --- | --- | --- | --- | --- | --- |
|  |  | |  | |  |
| Cc_4c | Chip colour after storage | | Chip colour after tubers were stored for 3 months at 4 ^o^C. 1= very dark brown to 10= very nice light golden colour | |  |
| Cc_4c_rec | Chip colour after storage and reconditioning | | Chip colour after cold storage at 4 ^o^C during 3 months and then reconditioning for 3 weeks at room temperature (18-20C). 1= very dark brown to 10= very nice light golden colour | |  |
| Cc_ah | Chip colour after harvest | | Chip colour after harvesting: 1= very dark brown to 10= very nice light golden colour. From 7 colour acceptable by industry | |  |
| Ccdif_4c_ah | Chip colour difference storage-harvest | | Difference in chip colour between cold storage and harvest. | |  |
| Ccdif_rec_4c | Chip colour difference recondition-storage | | Difference in chip colour between reconditioning and storage at 4 ^o^C. | |  |
| Ccdif_rec_ah | Chip colour difference recondition-harvest | | Difference in chip colour between reconditioning  and harvest. | |  |
| Discol_dif | Difference in discoloration | | Difference in enzymatic discoloration between 3 hours and 5 minutes | |  |
| Discol30min | Discoloration of flesh at 30 min | | Enzymatic discoloration of raw flesh, after 30 minutes: 0= no change in flesh colour until 6=very dark red/brown colour | |  |
| Discol3h | Discoloration flesh at 3h | | Enzymatic discoloration raw flesh after 3h From 0= no change in flesh colour until 8= black flesh colour | |  |
| Discol5min | Discoloration of flesh at 5 min | | Enzymatic discoloration of raw flesh, after 5 minutes: 0= no change in flesh colour until 6=very dark red/brown colour | |  |
| Discolcook_diff | Difference in discoloration after cooking | | Difference in discoloration after cooking between 24 and 5 minutes values | |  |
| Discolcook24h | Discoloration after cooking-24h | | Same as Discolcook5m | |  |
| Discolcook30min | Discoloration after cooking-30min | | Same as Discolcook5m | |  |
| Discolcook5m | Discoloration after cooking-5min | | Discoloration after cooking, after 5 minutes: 1= no change in colour; 2= some light grey spots; 3= some darker grey spots; 4= light grey colour evenly distributed on the tuber; 5= dark grey colour evenly distributed on the tuber; 6=very dark grey/black colour evenly distributed on the tuber | |  |
| Carbohydrate and starch metabolism related quality traits | | Description | | Scoring | |
| DSC_T_diff | | Differential Scanning Calorimetry | | Starch gelatinization properties. | |
| DSC_T_end | | Differential Scanning Calorimetry | | Starch gelatinization properties. End temperature of gelatinization | |
| DSC_T_onset | | Differential Scanning Calorimetry | | Starch gelatinization properties. Onset temperature of gelatinization | |
| DSC_T_peak | | Differential Scanning Calorimetry | | Starch gelatinization properties. Peak temperature of gelatinization | |
| DSCdH | | Starch gelling delta H | | Temperature difference | |
| Flesh | | Flesh colour | | Flesh colour: 1= white cream; 2= cream;3= cream orange;4=yellow cream; 5=light yellow; 6=medium yellow; 7=dark yellow; 8=medium orange; 9=dark orange | |
| PSD_d50 | | Particle_size_distribution midpoint | | median of the particle size distribution of starch particles obtained from tubers | |
| PSD_d90_d10 | | Particle_size_distribution d90-d10 | | Starch grain particle size distribution: difference between 90 percentile point and 10 percentile point (indicates distribution range) | |
| Spec_grav_starch | | Underwater weight derived dry matter content | | Specific gravity: (5000xdry weight)*underwater weight. For chips, between 400-450; for French fries, between 450 and 500 and for starch industry >500 | |
| % Amylose | | % amylose | | Amylose fraction of starch, in percentage | |
