# Supplementary material for: Genetical genomics of quality related traits in potato tubers using proteomics
Source: BMC Plant Biol. 2018 Jan 23;18:20. doi: 10.1186/s12870-018-1229-1 (PMC5781343; doi:10.1186/s12870-018-1229-1)
Supplement: Supplementary file 2 — pQTL analysis summary for 2002 and 2003 harvest. Number of significant protein QTLs is depicted as well as the chromosomes containing the highest and lowest number of pQTLs. (DOCX 12 kb) [file 12870_2018_1229_MOESM2_ESM.docx]

**Additional file 2(Table 2):** pQTL analysis summary for 2002 and 2003 harvest.

Number of significant protein QTLs is depicted as well as the chromosomes containing the highest and lowest number of pQTLs.

| Year of harvest | Nr. of sig. pQTLs | Highest nr. of pQTL position | Lowest nr. of pQTL position | Nr. of multiple pQTLs |
| --- | --- | --- | --- | --- |
|  |  |  |  |  |
| 2002 | 190 | Chrom 8 (41*) | Chrom 10 (2*) | 20 |
|  |  |  |  |  |
| 2003 | 173 | Chrom 8 (31*) | Chrom 10 (3*) | 17 |
|  |  |  |  |  |

* Indicated numbers of pQTLs in that chromosome
