# Supplementary material for: Genetical genomics of quality related traits in potato tubers using proteomics
Source: BMC Plant Biol. 2018 Jan 23;18:20. doi: 10.1186/s12870-018-1229-1 (PMC5781343; doi:10.1186/s12870-018-1229-1)
Supplement: Supplementary file 3 — Summary of Phenotypic QTL results of the various quality traits, their peak position and explained variance at peak position. (DOCX 14 kb) [file 12870_2018_1229_MOESM3_ESM.docx]

**Additional file 3 (Table 3)**: Summary of Phenotypic QTL results of the various quality traits, their peak position and explained variance at peak position

| Traits* | Year | Chr. | Peak LOD | QTL Peak Marker | QTL Peak Pos. | %Var(R2) |
| --- | --- | --- | --- | --- | --- | --- |
| GemDS | 2003 | 1 | 4.4 | PotSNP1016 | 78.2 | 18.0 |
| Starch_grT | 2002 | 1 | 4.4 | PotSNP558 | 126.7 | 19.3 |
| Decol3h | 1998 | 2 | 4.4 | PotSNP133 | 52.9 | 21.6 |
| DSC_T_end | 2002 | 2 | 8.7 | PotSNP128 | 62.6 | 37.1 |
| %Amylose | 2002 | 2 | 5.4 | PotSNP108 | 73.7 | 25.1 |
| %Amylose | 2003 | 2 | 5.0 | PotSNP108 | 73.7 | 23.8 |
| DSC_T_onset | 2002 | 2 | 8.0 | POCI_38201 | 80.2 | 35.7 |
| DSC_T_peak | 2002 | 2 | 5.9 | POCI_38201 | 80.2 | 28.3 |
| Starch_Phos | 2002 | 2 | 6.9 | POCI_38201 | 80.2 | 31.8 |
| DSC_T_onset | 2003 | 2 | 14.8 | POCI_38201 | 80.2 | 55.7 |
| DSC_T_end | 2003 | 2 | 12.4 | POCI_38201 | 80.2 | 50.2 |
| DSC_peak | 2003 | 2 | 12.0 | POCI_38201 | 80.2 | 48.7 |
| Starch_Phos | 2003 | 2 | 6.5 | POCI_38201 | 80.2 | 30.1 |
| Decol_diff | 1998 | 3 | 4.5 | PotSNP37M | 40.1 | 18.7 |
| Flesh | 1998 | 3 | 10.5 | PotSNP502 | 78.5 | 44.3 |
| Decol5min | 1998 | 3 | 5.5 | PotSNP1001 | 81.4 | 26.4 |
| Decol30min | 1998 | 3 | 5.5 | PotSNP1001 | 81.4 | 26.5 |
| DSC_T_peak | 2002 | 5 | 4.5 | PotSNP125 | 23.6 | 21.2 |
| Cc_4c | 1998 | 5 | 5.7 | PotSNP125 | 23.6 | 27.4 |
| Starch_Phos | 2003 | 5 | 6.6 | PotSNP621 | 44.3 | 30.8 |
| PSD_d9_d10 | 2002 | 6 | 5.0 | PotSNP963 | 56.4 | 21.2 |
| Spec_grav_starch | 2002 | 8 | 4.4 | PotSNP12 | 67.8 | 21.1 |
| Spec_grav_starch | 2002 | 10 | 5.1 | PotSNP76 | 26.8 | 24.7 |

*Please find the explanation of these abbreviations and how the trait was scored in the supplementary table 1
