# Supplementary material for: Genetical genomics of quality related traits in potato tubers using proteomics
Source: BMC Plant Biol. 2018 Jan 23;18:20. doi: 10.1186/s12870-018-1229-1 (PMC5781343; doi:10.1186/s12870-018-1229-1)
Supplement: Supplementary file 4 — Co-localization of phQTLs (starch, colour and cold sweetening related traits) and pQTLs for proteomics data in 2002. (DOCX 13 kb) [file 12870_2018_1229_MOESM4_ESM.docx]

**Additional file 4 (Table 4)**: Co-localization of phQTLs (starch, colour and cold sweetening related traits) and pQTLs for proteomics data in 2002

| Traits | Chr. Nr. | QTL Peak |
| --- | --- | --- |
|  |  | Pos. (cM) |
| Starch_grT_2002 | 1 | 126.7 |
| Pro_379 | 1 | 135.8 |
| Pro_102 | 1 | 135.8 |
| % Amylose_2003 | 2 | 73.7 |
| %Amylose_2002 | 2 | 73.7 |
| DSC_T_onset_2002 | 2 | 80.2 |
| DSC_T_peak_2002 | 2 | 80.2 |
| Starch_Phos_2002 | 2 | 80.2 |
| DSC_T_onset_03 | 2 | 80.2 |
| DSC_T_end_2003 | 2 | 80.2 |
| DSC_T_peak_2003 | 2 | 80.2 |
| Starch_Phos_2003 | 2 | 80.2 |
| Pro_169 | 2 | 80.2 |
| Discol3h | 2 | 52.9 |
| DSC_T_end_2002 | 2 | 62.6 |
| Pro_736 | 2 | 49.3 |
| Discol_diff | 3 | 40.1 |
| Pro_1282 | 3 | 40.1 |
| Discol5min | 3 | 81.4 |
| Discol30min | 3 | 81.4 |
| Flesh colour | 3 | 78.5 |
| Pro_997 | 3 | 78.5 |
| Pro_64 | 3 | 78.5 |
| Pro_943 | 3 | 78.5 |
| Pro_1000 | 3 | 78.5 |
| Pro_1129 | 3 | 80.8 |
| Pro_1245 | 3 | 80.8 |
| Pro_1266 | 3 | 80.8 |
| Pro_1391 | 3 | 80.8 |
| Pro_1297 | 3 | 80.8 |
| Pro_1240 | 3 | 80.8 |
| Pro_1024 | 3 | 80.8 |
| Pro_1021 | 3 | 80.8 |
| Pro_6 | 3 | 85.5 |
| Pro_152 | 3 | 85.5 |
| DSC_T_peak_2002 | 5 | 23.6 |
| Cc_4c | 5 | 23.6 |
| Pro_1045 | 5 | 23.6 |
| Pro_1035 | 5 | 23.6 |
| Starch_Phos_2003 | 5 | 44.3 |
| Pro_129 | 5 | 40.3 |
| Pro_128 | 5 | 40.3 |
| Pro_142 | 5 | 48.2 |
| Pro_848 | 5 | 48.2 |
| Pro_143 | 5 | 48.9 |
| Pro_144 | 5 | 53.3 |
| Pro_140 | 5 | 54.8 |
| Pro_150 | 5 | 54.8 |
| Pro_928 | 5 | 54.8 |
| PSD_d9_d10_2002 | 6 | 56.4 |
| Pro_254 | 6 | 56.4 |
| Pro_251 | 6 | 56.4 |
| Pro_255 | 6 | 60.0 |
| Spec_grav_starch | 8 | 67.8 |
| Pro_275 | 8 | 59.2 |
