# Supplementary material for: Genetical genomics of quality related traits in potato tubers using proteomics
Source: BMC Plant Biol. 2018 Jan 23;18:20. doi: 10.1186/s12870-018-1229-1 (PMC5781343; doi:10.1186/s12870-018-1229-1)
Supplement: Supplementary file 5 — Co-localization of phQTLs (starch, colour and cold sweetening related traits) and pQTLs for proteomics data in 2003. (DOCX 25 kb) [file 12870_2018_1229_MOESM5_ESM.docx]

**Additional file 5 (Table 5)**: Co-localization of phQTLs (starch, colour and cold sweetening related traits) and pQTLs for proteomics data in 2003

| Traits | Chr. Nr. | QTL Peak |
| --- | --- | --- |
|  |  | Pos. (cM) |
| Starch_grT_2002 | 1 | 126.7 |
| Pro_1240 | 1 | 134.6 |
| DSC_T_onset_2002 | 2 | 80.2 |
| DSC_peak_2002 | 2 | 80.2 |
| Starch_Phos_2002 | 2 | 80.2 |
| DSC_T_onset_2003 | 2 | 80.2 |
| DSC_T_end_2003 | 2 | 80.2 |
| DSC-T_peak_2003 | 2 | 80.2 |
| Starch_Phos_2003 | 2 | 80.2 |
| % Amylose_2002 | 2 | 73.7 |
| % Amylose_2003 | 2 | 73.7 |
| Pro_152 | 2 | 73.7 |
| Pro_188 | 2 | 73.7 |
| Pro_272 | 2 | 80.2 |
| Flesh colour | 3 | 78.5 |
| Discol5min | 3 | 81.4 |
| Discol30min | 3 | 81.4 |
| Pro_1007 | 3 | 74.0 |
| Pro_1536 | 3 | 74.0 |
| Pro_1269 | 3 | 74.0 |
| Pro_1270 | 3 | 74.0 |
| Pro_1160 | 3 | 74.0 |
| Pro_971 | 3 | 74.0 |
| Pro_951 | 3 | 74.0 |
| Pro_491 | 3 | 74.0 |
| Pro_1217 | 3 | 78.5 |
| Pro_1416 | 3 | 78.5 |
| Pro_1021 | 3 | 78.5 |
| Pro_943 | 3 | 78.5 |
| Pro_1217 | 3 | 78.5 |
| Pro_1416 | 3 | 78.5 |
| Pro_943 | 3 | 78.5 |
| Pro_1129 | 3 | 80.8 |
| Pro_1245 | 3 | 80.8 |
| Pro_1091 | 3 | 80.8 |
| Pro_1267 | 3 | 80.8 |
| Pro_1000 | 3 | 80.8 |
| Pro_1297 | 3 | 80.8 |
| Pro_1240 | 3 | 80.8 |
| Pro_1318 | 3 | 80.8 |
| Pro_1391 | 3 | 80.8 |
| Pro_1272 | 3 | 80.8 |
| Pro_64 | 3 | 82.5 |
| Pro_1317 | 3 | 82.5 |
| Pro_152 | 3 | 86.4 |
| Pro_153 | 3 | 86.4 |
| Pro_1294 | 3 | 88.5 |
| DSC_T_peak_2002 | 5 | 23.6 |
| Cc_4c | 5 | 23.6 |
| Pro_1438 | 5 | 23.6 |
| Pro_366 | 5 | 23.6 |
| Pro_469 | 5 | 23.6 |
| Pro_339 | 5 | 23.6 |
| Pro_41 | 5 | 23.6 |
| Pro_330 | 5 | 23.6 |
| Pro_192 | 5 | 23.6 |
| Pro_36 | 5 | 23.6 |
| Pro_40 | 5 | 23.6 |
| Pro_1051 | 5 | 23.6 |
| Pro_39 | 5 | 23.6 |
| Pro_357 | 5 | 23.6 |
| Pro_666 | 5 | 20.1 |
| Pro_1264 | 5 | 20.1 |
| Pro_1317 | 5 | 18.4 |
| Starch_Phos_2003 | 5 | 44.3 |
| Pro_140 | 5 | 51.5 |
| Pro_144 | 5 | 44.8 |
| Pro_150 | 5 | 44.8 |
| Pro_128 | 5 | 42.3 |
| Pro_129 | 5 | 40.3 |
| PSD_d9_d10_2002 | 6 | 56.4 |
| Pro_251 | 6 | 56.4 |
